# Knockdown of SDC-1 Gene Alleviates the Metabolic Pathway for the Development of MODS

**DOI:** 10.1007/s12033-023-00809-9

**Published:** 2023-07-29

**Authors:** Jiangang Xie, Yuexiang Ma, Yang Huang, Qianmei Wang, Yunyun Xu, Qi Zhang, Jing Yang, Wen Yin

**Affiliations:** Department of Emergency, Xijing Hospital, Air Force Medical University, Xi’an, 710032 Shaanxi China

**Keywords:** Syndecan-1, Nicotinamide, Nicotinamide metabolism, MODS

## Abstract

This study aims to reveal the metabolic differences between SDC-1 knockout mice and wild-type mice and the metabolic differences caused by shock in SDC-1 knockout mice by integrating transcriptomics and metabolomics. A total of 1009 differential metabolites were differentially expressed based on untargeted metabolomics and high-resolution mass spectrometry detection techniques. According to Kyoto Encyclopedia of Genes and Genomes enrichment, SDC-1 knockout significantly altered fat digestion and absorption, GnRH signaling pathway, fructose and mannose metabolism, and some other amino-related metabolic pathways and significantly modulated positively regulated longevity regulatory pathways, longevity regulatory pathways-worm, nicotinamide and niacinamide metabolism, and vitamin digestion and absorption pathways after its shock. Our findings indicate that SDC-1 knockout may have potential therapeutic effects in hemorrhagic shock by increasing nicotinamide metabolism.

**Supplementary Information:**

The online version contains supplementary material available at 10.1007/s12033-023-00809-9.

## Information

Hemorrhagic shock as the leading cause of death worldwide and, with an aging population, injury mortality will continue to rise [1, 2]. According to the World Health Organization’s mortality database (2020), deaths due to traumatize increase with age from 3089 (0–5 years) to 50 890 (> 75 years) [3]. The imbalance between pro-inflammatory and anti-inflammatory effects as an important component of the body's immune system function leads to the main cause of death from trauma [4]. In the early stages of the inflammatory process, the body’s immune system produces a strong pro-inflammatory effect [5]. As the disease progresses, the immune system gradually shifts from a pro-inflammatory to an anti-inflammatory state in the early stages. When the balance between pro-inflammation and anti-inflammation is disrupted, it leads to an inflammatory storm and immunosuppression, which results in excessive cellular damage and ultimately multi-organ dysfunction syndrome (MODS) [5, 6]. MODS as the most serious complication of sepsis is still unclear as to the regulatory mechanism. The clinical treatment is still based on antibiotics and organ support, but the treatment is not satisfactory [7, 8]. Approximately half of all critically ill patients in the ICU develop multiple organ dysfunction syndrome (MODS), which is responsible for 30% of deaths worldwide [9, 10]. Therefore, exploring the specific regulatory mechanisms of MODS occurrence after trauma is key to reducing MODS mortality.

The balance of pro- and anti-inflammatory cellular metabolism is key to the treatment of MODS. The extracellular matrix (ECM), a structure formed around cells, not only provides tissue dynamics and integrity, but also provides its own right signaling molecules involved in driving many biological functions, such as cell migration, survival, and metabolism [11]. The vascular endothelial glycocalyx is the first ECM structure to which immune cells are exposed, and the glycocalyx at the site of injury induces an injury-associated immune response, releasing inflammatory factors and leading to immune imbalance. This imbalance of immune homeostasis in excessive inflammation and cellular storm leads to the emergence of pathological states [12–14]. Thus, the regulation of tissue immune homeostasis involves complex immune-stromal cross-talk that is influenced by the extracellular matrix (ECM) [14, 15]. The main components of the extracellular matrix (ECM) are syndecan-1 (SDC-1), which is bound to the cell membrane, and membrane-bound glycosaminoglycans (GAGs), such as heparan sulfate (HS), chondroitin sulfate (CS), and hyaluronic acid (HA) [16, 17]. As the main core protein of the glycoprotein chain, SDC-1 is a glycocalyx degradation product after vascular endothelial cell injury in trauma patients. The accumulation of matrix metalloproteinases, thrombin, and fibrin under inflammatory conditions accelerate the shedding of SDC-1 from the endothelial surface [18–21]. However, the role of SDC-1 in the development of MODS has not yet been investigated.

## Experiential Section

### Mice

Male C57BL/6 mice of specific pathogen-free grade were purchased from the animal center of the Air Force Medical University. The mice were used at the ages of 8 to 12 weeks and weighing between 20 and 25 g. They were randomly assigned to the control and MODS group. The sequence of primer pairs was as follows (5′ → 3′, Forward Primer and Reverse Primer): SDC-1: CTGCCGCAAATTGTGGCTAC and TGAGCCGGAGAAGTTGTCAGA; GAPDH: GGAGCGAGATCCCTCCAAAAT and GGCTGTTGTCATACTTCTCATGG. Both the WT and SDC-1 groups of mice were anesthetized using inhalation of isoflurane (RWD Life Science, San Diego, CA, USA) through a small animal anesthesia ventilator (Shenzhen Deweida Technology Co., Ltd., China). Mice in the WT shock and SDC-1 shock groups were similarly anaesthetized and 30% of the blood volume (calculated as: 0.6 ml for a 25 g mice) was collected from the heart within 60 s to establish the MODS model following a previous protocol [22]. All mice were euthanized 2 h after a cardiac puncture. All experiments were conducted in compliance with the guide for the care and use of laboratory animals and approved by the Institutional Animal Care and Use Committee of the Air Force Medical University.

### Sample Preparation for Nontargeted LC–MS Analysis

The plasma samples were thawed at 4 ℃ and 100 μL aliquots were mixed with 400 μL cold methanol/acetonitrile (1:1, *v*/*v*) to remove the protein. The mixture was centrifuged for 15 min (14,000 g, 4 ℃). The samples were redissolved in 100 μL acetonitrile/water (1:1, *v*/*v*) solvent and subjected to LC–MS analysis.

### LC–MS/MS Analysis

An Agilent 1290 Infinity UHPLC-MS system coupled to an AB Triple TOF 6600 mass spectrometer was used to conduct plasma metabolite analysis. The ACQUITY UPLC BEH Amide was employed for chromatographic separation (2.1 mm × 100 mm, 2.5 μm, Waters) [23, 24]. MS was performed in both ion mode and a top-10 scan mode. In ESI-positive mode, the mobile phase contained A water with 0.1% formic acid and B acetonitrile with 0.1% formic acid, and in ESI-negative mode, the mobile phase contained A 0.5 mM ammonium fluoride in water and B acetonitrile. The gradient was 1% B for 1.5 min and was linearly increased to 99% in 11.5 min and kept for 3.5 min. The reduction was accomplished within 0.1 min, bringing it down to 1%, followed by a re-equilibration period of 3.4 min. The gradients were maintained at a flow rate of 0.3 mL/min, and the column temperature was held constant at 25 ℃. A 2 μL sample aliquot was then injected. The ESI source conditions were set as follows: ion source gas 1 as 60, ion source gas 2 as 60, curtain gas as 30, source temperature: 600 ℃, and ion spray voltage floating (ISVF) ± 5500 V. In MS only acquisition, time for TOF MS scan was set at 0.2 s/spectra. In auto-MS/MS acquisition, the instrument was set to acquire m/z at the range of 25–1000 Da, and the accumulation time for product ion scan was set at 0.05 s/spectra over the m/z range of 25–1000 Da, and the accumulation time for product ion scan was set at 0.05 s/spectra. Information-dependent acquisition (IDA) was employed to obtain the production ion scan, with high sensitivity mode chosen. The parameters were set as follows: the collision energy (CE) was fixed at 35 V with ± 15 eV; declustering potential (DP), 60 V ( +) and 60 V (−); exclude isotopes within 4 Da, candidate ions to monitor per cycle: 10.

### Date Analysis

Data were analyzed and processed and plotted using recovery component (v2.18.1) and origin software (v10.0.0.154) [25, 26]. Significance was determined using the Student’s *t* test, and molecular features were selected at a *p* value of < 0.05. Pathway analysis was performed on the Kyoto Encyclopedia of Genes and Genomes platform (https://www.kegg.jp/kegg/pathway.html).

## Results

### Mice Characteristics and Reproducibility

**Fig. 1:**
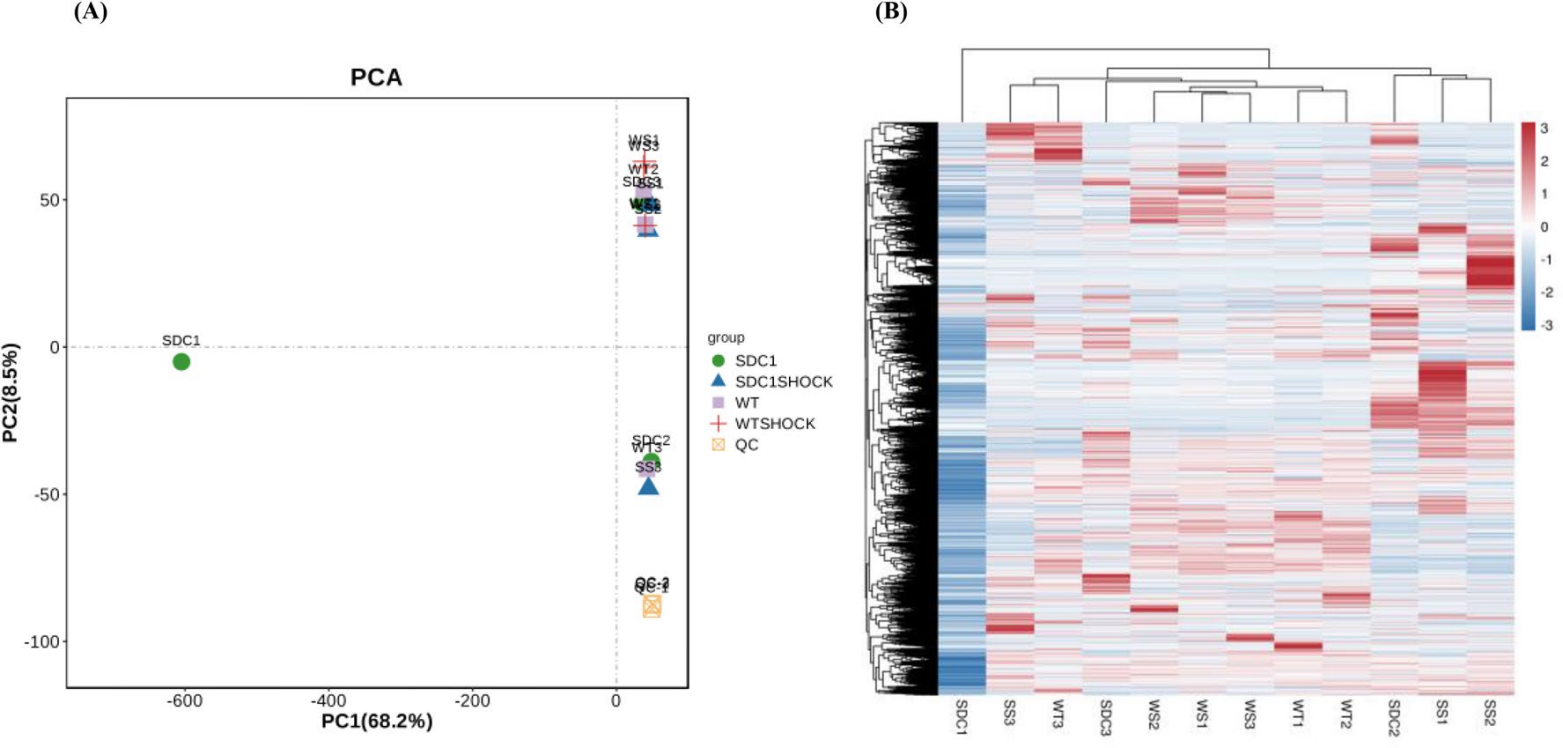
**A** PCA score plot of metabolome from SDC-1 knockout, SDC-1 knockout shock, WT, and control mice for 8–12 weeks and pooled QCs. The pooled QC group (in pink) clearly demonstrates that the instrument variability is low across the run. **B** Clustering heat map by the samples. Four groups are included

The SDC-1 knockout, SDC-1 knockout shock, WT shock, and control mice (WT) were carefully matched based on age and sex. Genotyping was performed to verify the SDC-1 knockout, as depicted in Fig. 1A. The reproducibility of our metabolomic analysis method was assessed using quality control (QC) samples inserted in the sequence. QCs were tightly clustered in the principal component analysis (PCA) score plot as presented in Fig. 1A. The retention time fluctuation was lower than 0.2 min, and the RSD values of peak intensities were < 10%. These results indicated that our metabolomic method was stable and reliable. The analysis of metabolite clustering revealed significant differences between the metabolites found in WT-type mice after shock and those found in SDC-1 knockout mice after shock. The typical full-scan base peak ion chromatograms of SDC-1 knockout mice and controls in positive ion mode are presented in Figure S1.

### Multivariate Data Analysis

**Fig. 2:**
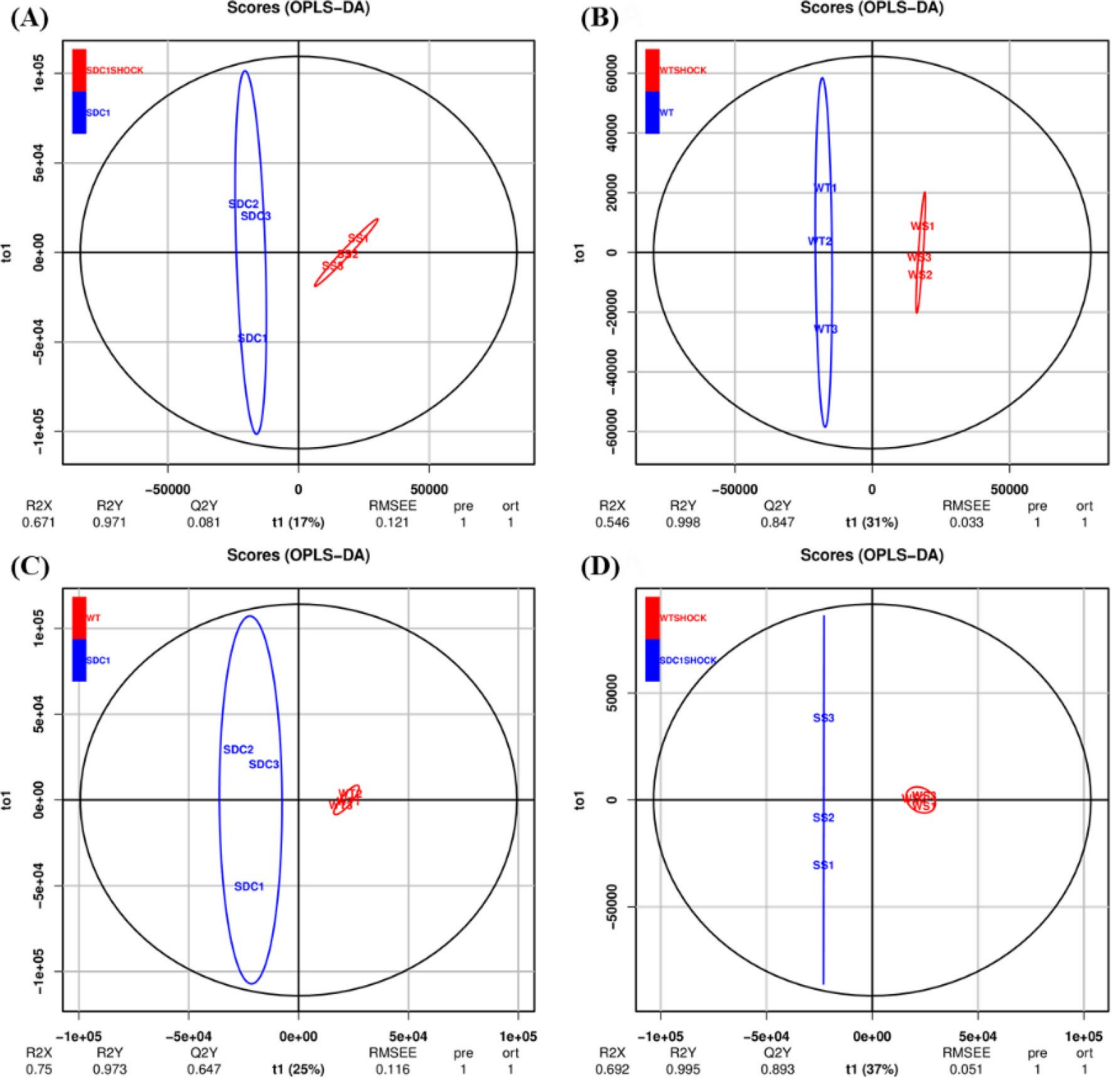
OPLS-DA score plot of plasma metabolites after administration of WT and WT shock, SDC-1 gene knockout and shock. **A** SDC-1 *vs* SDC-1 shock; **B** WT *vs* WT shock; **C** WT *vs* SDC-1; **D** WT shock *vs* WT SDC-1

OPLS-DA was performed on all analyzed metabolites to compare differences in plasma metabolism among WT, WT shock, SDC-1 knockout, and SDC-1 knockout shock mice. It can be inferred that the R^2^X and R^2^Y of SDC-1 knockout and SDC-1 shock mice were 0.671 and 0.971, the R^2^X and R^2^Y of WT and WT shock mice were 0.546 and 0.998, and the R^2^X and R^2^Y of WT and SDC-1 were 0.75 and 0.973, respectively (Fig. 2). The R^2^X and R^2^Y of WT shock and SDC-1 shock mice were 0.692 and 0.995, respectively (Fig. 2). The metabolic profiles of the four types of mice were markedly distinct and showed significant differences between groups, while within-group differences were not significant. These observations suggest that the metabolite models fit well and were able to accurately differentiate between the groups. These results showed that the four models from each of the two models (SDC-1 knockout, SDC-1 knockout shock, WT, or WT shock) were clustered together, and the two groups were could be separated effectively, suggesting that the models were successfully established.

### Differential Metabolite Analysis

**Fig. 3:**
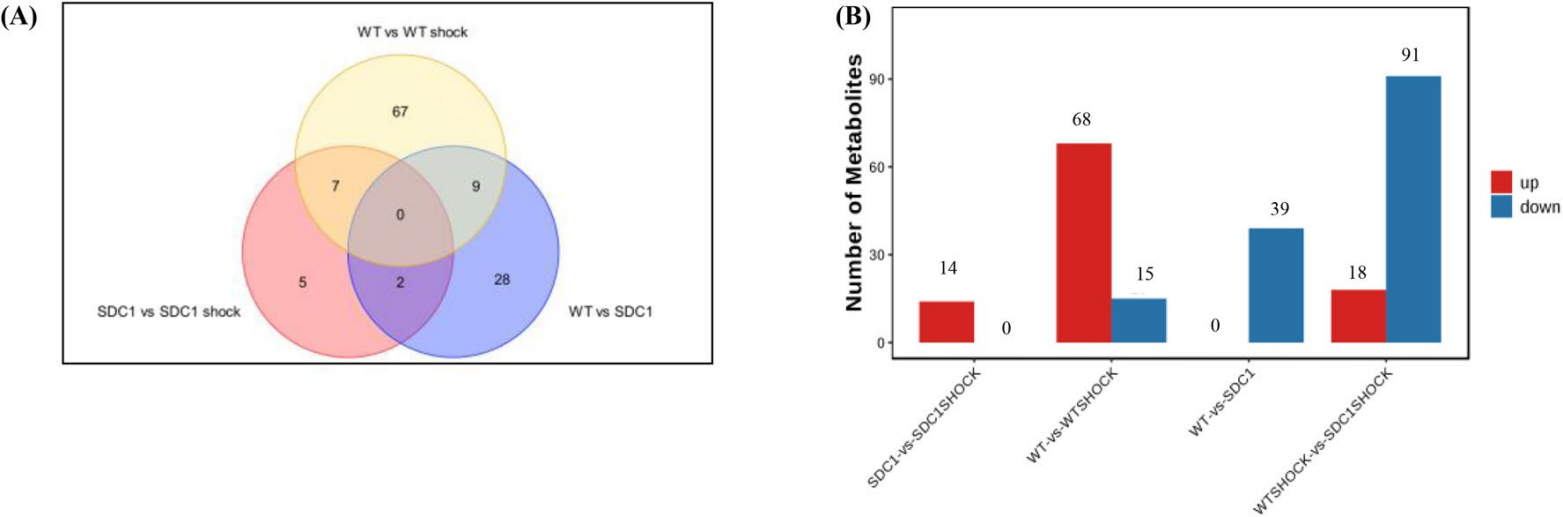
Statistical chart of differences in each group. **A** Venn diagram of the number of DMs of WT *vs* SDC-1 gene knockout, WT shock *vs* SDC-1 gene knockout shock and WT *vs* WT shock mice; **B** Number of metabolites up- and down-regulated in plasma revealed by pairwise comparisons. Metabolites with VIP ≤ 1.0 and *p* < 0.05 were selected as significant differences

In all four experimental groups, each of which was repeated five times, all metabolites demonstrated significant differences at VIP ≥ 1.0 and *p* < 0.05 (Fig. 3 and Table 1), indicating remarkable differences among the groups in terms of metabolism. When compared to SDC-1 gene knockout, the plasma samples of WT and SDC-1 gene knockout shock revealed a varying number of differential metabolites (DMs). 39 DMs were identified to be down-regulated in WT, while 14 DMs were up-regulated in SDC-1 gene knockout. Notably, compared to the WT, 109 differential metabolites (18 up-regulated and 91 down-regulated) were identified in the plasma samples of SDC-1 gene knockout shock, while 83 differential metabolites (68 up-regulated and 15 down-regulated) were found in WT shock. These findings suggest that these metabolites may be involved in the up-regulation of SDC-1, when it is knocked out, and in the down-regulation of metabolites during shock, which may have a better alleviating effect on shock. Interestingly, WT shock exacerbates the shock response by up-regulating metabolic pathways. Our hypothesis is that SDC-1 gene knockout may have contributed to negative regulation of the metabolic pathway, thereby attenuating the shock response.

**Table 1:** KEGG different metabolic pathways ID and the proportion of different metabolic pathways between WT shock *vs* SDC-1 shock groups (VIP ≥ 1.0 and *p* < 0.05)

Pathway	All metabolites with pathway annotation (455)	*p* value	Pathway ID	C_id desc
Histidine metabolism	15 (3.3%)	0.006071	ko00340	Carnosine; nalpha-(beta-alanyl)-*L*-histidine
				*N*(pi)-methyl-*L*-histidine; *N*-pros-methyl-*L*-histidine; 3-methylhistidine; etc
				Beta-alanyl-*N*-methyl-*L*-histidine; anserine
				Imidazole-4-acetate; imidazoleacetic acid; 4-imidazoleacete
				Ergothionenine
Vitamin digestion and absorption	16 (3.52%)	0.008773	ko04977	Nicotinamide; nicotinic acid amide; niacinamide; vitamin PP
				Cholesterol; cholest-5-*en*-3beta-ol
				Pantothenate; pantothenic acid; (*R*)-pantothenate
				Carnosine; nalpha-(beta-alanyl)-*L*-histidine
				Beta-alanyl-*N*-methyl-*L*-histidine; anserine
				Thiamine; vitamin B1; aneurin; antiberiberi factor
Beta-alanine metabolism	10 (2.2%)	0.026139	ko00410	Carnosine; Nalpha-(beta-alanyl)-*L*-histidine
				Pantothenate; pantothenic acid; (R)-pantothenate
				beta-Alanyl-*N*-methyl-*L*-histidine; anserine
Retrograde endocannabinoid signaling	10 (2.2%)	0.026139	ko04723	Phosphatidylcholine; lecithin; phosphatidyl-*N*-trimethylethanolamine; etc
				2-Arachidonoylglycero; 2-arachidonoyl-sn-glycerol; 2-AG
				Diacylglycerol; diglyceride
Fat digestion and absorption	6 (1.32%)	0.028354	ko04975	Diacylglycerol; diglyceride
				Cholesterol; cholest-5-*en*-3beta-ol
				Phosphatidate; phosphatidic acid; 1,2-diacyl-*sn*-glycerol-3-phosphate; 3-*sn*-phosphatidate
Longevity-regulating pathway worm	3 (0.66%)	0.042605	ko04212	Nicotinamide; nicotinic acid amide; vitamin PP
				Nicotinamide; nicotinic acid amide; vitamin PP

### KEGG Metabolic Pathway Analysis

**Fig. 4:**
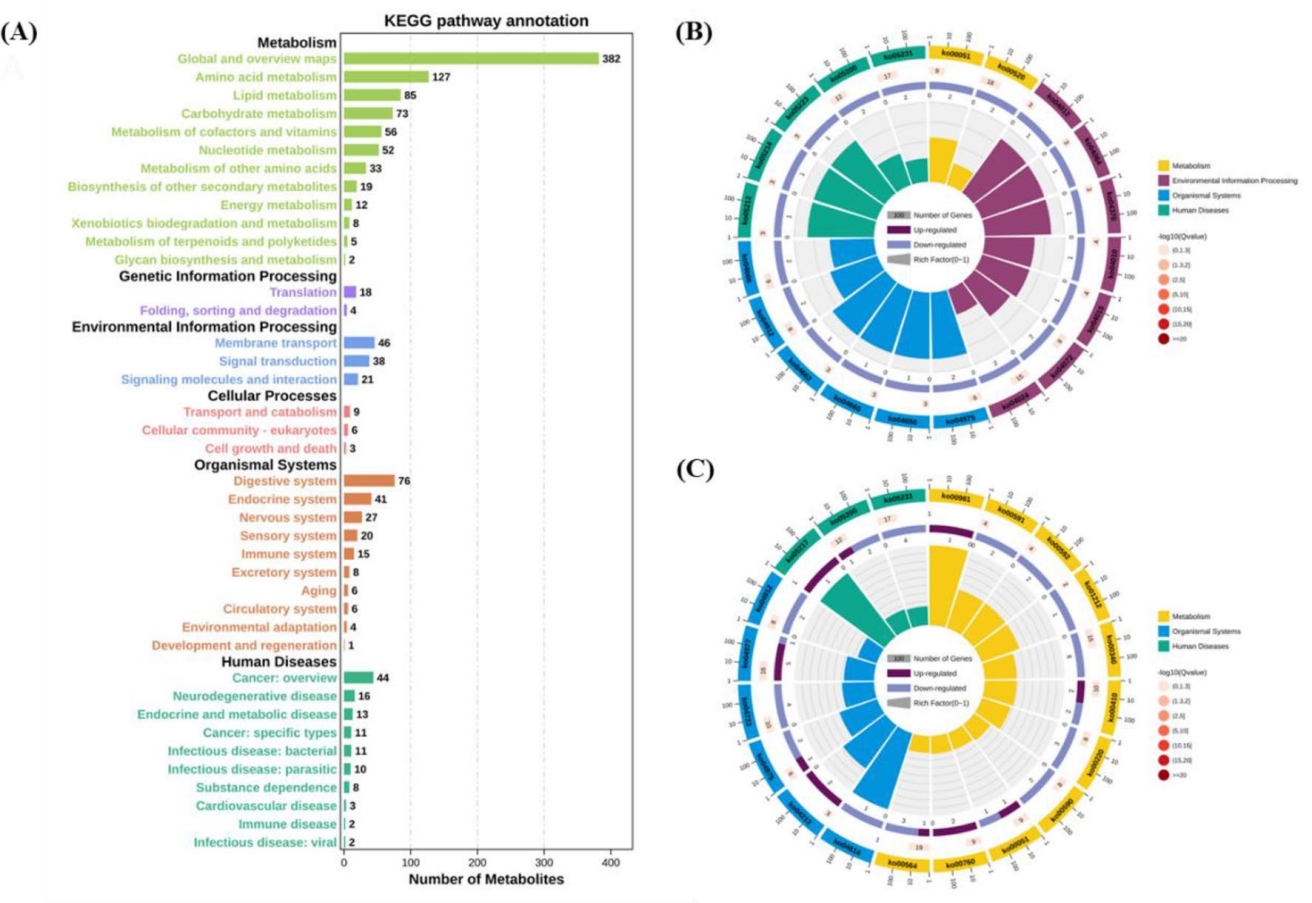
Diagram of metabolic pathway analysis. **A** KEGG pathway annotation of metabolites; **B** KEGG enrichment analysis of WT *vs* SDC-1 gene knockout differential metabolites; **C** KEGG enrichment analysis of WT shock *vs* SDC-1 gene knockout shock. Metabolic pathways with VIP ≥ 1.0 and *p* < 0.05 were selected as significant differences

The KEGG statistical maps revealed that in WT, WT shock, SDC-1 gene knockout, and shock, the enriched pathways were those associated with the regulation of metabolic pathways and organismal systems. Among the metabolic pathways, the top three pathways in which metabolites were as follows: global and overview maps, amino acid metabolism, and lipid metabolism (Fig. 4A). The top 20 enriched pathways were selected for analysis based on the KEGG enrichment map. WT mice were involved in the positive regulation of fatty acid metabolism, propionate metabolism, pyrimidine metabolism, cellular processes and human disease pathways after shock. SDC-1 knockout mice regulate metabolism by negatively modulating fat digestion and absorption, GnRH signaling pathway, and fructose and mannose metabolism. Compared to plasma samples from shocked WT mice, plasma samples from SDC-1 knockout shocked mice show some degree of organismal regulation by participating in metabolic, organismal systems, and human disease pathways. All metabolites, except for nicotinamide, niacinamide, and rotundine, were negatively regulated. Further studies revealed that nicotinamide/niacinamide may be involved in the regulation of metabolism by altering the digestive system, aging, metabolism of cofactors, and vitamins pathways. Therefore, our hypothesis is that in response to shock, SDC-1 knockout mice positively regulate certain compounds such as nicotinamide and nicotinic acid, which are involved in the longevity-regulating pathway, nicotinate and nicotinamide metabolism, and vitamin digestion and absorption pathways (Fig. 4B, C and Table 2).

**Table 2:** KEGG different metabolic pathways ID and the proportion of different metabolic pathways between WT *vs* SDC-1 groups (VIP ≥ 10.0 and *p* < 0.05)

Pathway	All metabolites with pathway annotation (455)	*p* value	Pathway ID	C_id desc
Fat digestion and absorption	6 (1.32%)	0.016037	ko04975	Diacylglycerol; diglyceride
				Phosphatidate; phosphatidic; 3-*sn*-phosphatidate; etc
GnRH signaling pathway	8 (1.76%)	0.028721	ko04912	Diacylglycerol; diglyceride
				Phosphatidate; phosphatidic; 3-*sn*-phosphatidate; etc
Fructose and mannose metabolism	9 (1.98%)	0.036171	ko00051	*D*-Mannose; 1-phosphatidic acid; alpha-*D*-mannose 1-phosphate
				6-Deoxy-*L*-galactose; *L*-Fucose
Phospholipase D signaling pathway	9 (1.98%)	0.036171	ko04072	Diacylglycerol; diglyceride
				Phosphatidate; phosphatidic; 3-*sn*-phosphatidate, etc
Fc gamma R-mediated phagocytosis	9 (1.98%)	0.036171	ko04666	Diacylglycerol; diglyceride
				Phosphatidate; phosphatidic; 3-*sn*-phosphatidate; etc

## Discussion

Recently, application of metabolomics-based approaches have provided new insights into the development of disease [27, 28]. However, information on metabolic changes from SDC-1 knockout to shock is still scarce [29, 30]. Here, we detected the metabolome by using UHPLC-MS to detect mouse plasma. The results clearly demonstrated that the metabolites after shock in WT-type mice were significantly different from those in SDC-1 knockout mice after shock. Compared with WT mice, SDC-1 knockout mice show 18 up-regulated metabolites and 91 down-regulated metabolites. According to KEGG metabolic pathway analysis, the metabolism of nicotinamide/niacinamide in shock mice with gene knockout SDC-1 may be positively regulated by regulating the digestive system, aging, metabolism of cofactors, and vitamins pathways. In this study, we punctured the heart to obtain 30% of blood at 60 s to establish the MODS model, and metabolomics of four groups were determined using UHPLC-MS. We uncovered that the SDC-1 knockout mice showed a distinct metabolomic profiling compared with control. Enrichment and pathway analysis revealed that nicotinamide/niacinamide, tetrahydrogambogic acid, fructoselysine, and sesamin biosynthesis were significantly elevated when shock occurred after SDC-1 knockdown.

**Fig. 5:**
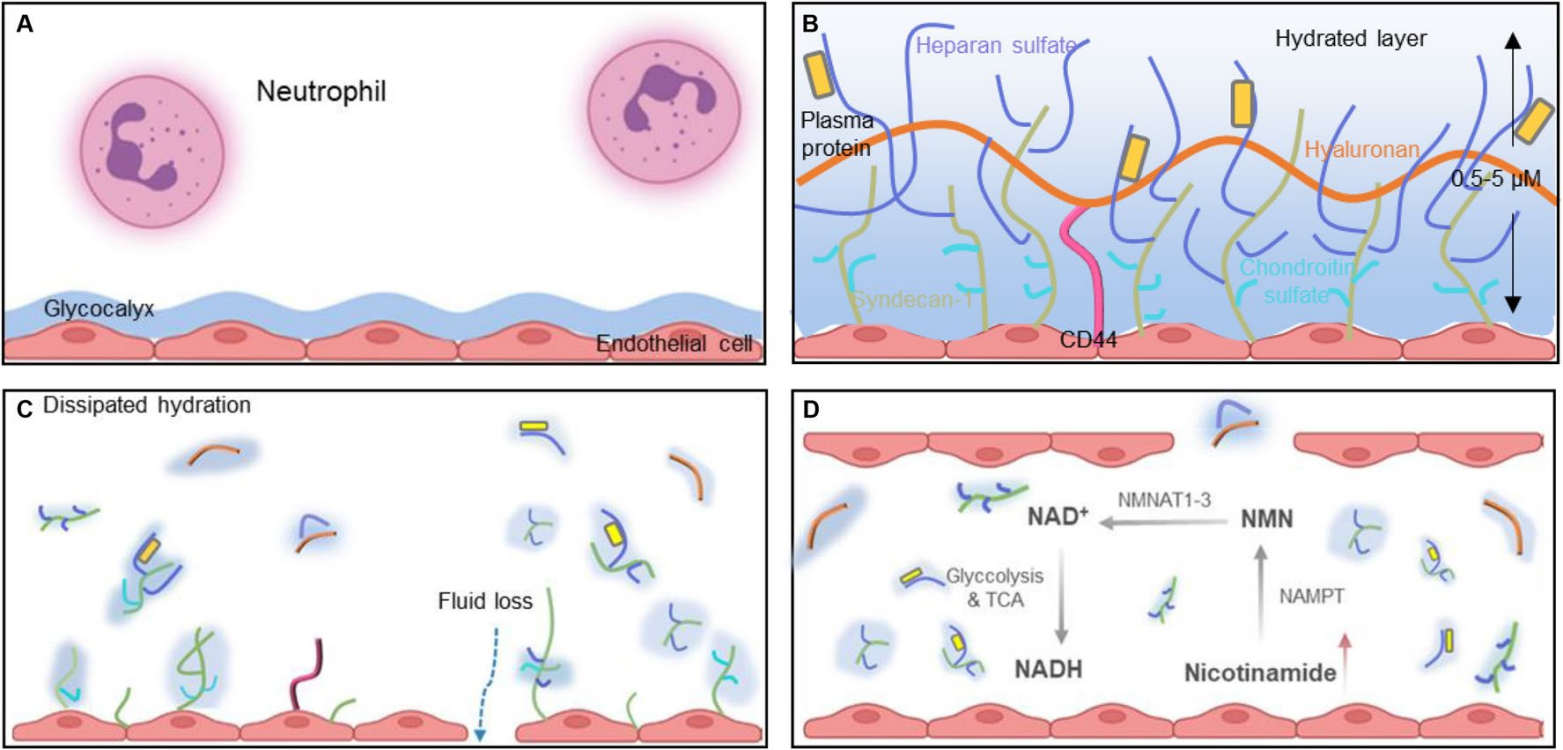
Regulation of metabolic pathways in SDC-1 knockout mice after shock

The tissue concentration of NAD^+^ rapidly decreases during hemorrhagic shock, which can lead to mitochondrial dysfunction and cell death [2]. According to the KEGG metabolic pathway analysis, compounds such as nicotinamide, niacinamide, and fructoselysine were elevated after shock in SDC-1 knockout mice compared to WT-type shock mice. The tissue concentration of NAD + rapidly decreases during hemorrhagic shock, which can lead to mitochondrial dysfunction and cell death. The degree of decrease in NAD^+^ concentration is directly proportional to the severity of the injury [31, 32]. Nicotinamide is converted to nicotinamide mononucleotide (NMN) in a rate-limiting step catalyzed by the enzyme nicotinamide phosphoribosyltransferase (NAMPT). NMN is then converted to NAD^+^ by 1 of 3 NMN adenylyltransferase isoforms (NMNAT1-3). NMN, a synthetic precursor of NAD^+^, significantly reduces inflammation, improves cellular metabolism, and increases survival after hemorrhagic shock [33–35]. Therefore, we postulate that SDC-1 may have therapeutic effects on hemorrhagic shock by increasing nicotinamide after shock, which elevates tissue NAD + levels, maintains cellular redox ratios, and enhances glycolysis and tricarboxylic acid cycle pathways. In addition, nicotinamide increases monomeric NMN which exerts anti-inflammatory effects, attenuating the systemic inflammatory response as well as the cellular effects of cytokine exposure (Fig. 5) [36, 37]. Given that nicotinamide is involved in cellular energy metabolism, signal transduction, and a range of inflammatory biochemical responses, it is a potential therapeutic target for the treatment of hemorrhagic shock.

## Conclusion

In summary, metabolomic analysis of SDC-1 knockout and hemorrhagic shock metabolites was investigated. The information supports the notion that the absence of SDC-1 in mice triggers an increase in nicotinamide metabolism following a shock event, by controlling pathways related to the digestive system, aging, and the metabolism of cofactors and vitamins. As a result, these pathways play a role in enhancing survival rates in response to hemorrhagic shock. Importantly, we found that when SDC-1 knockout mice were shocked, nicotinamide metabolites increased although other metabolic pathways decreased, which in turn improved mitochondrial function, inflammatory status, and lactate metabolism while maintaining cellular redox ratios and also enhanced the physiological resilience of the whole organism during hemorrhagic shock. This enhanced the physiological reserve and improved the survival of mice under hemorrhagic shock. Therefore, SDC-1 is expected to be a new therapeutic target. Considering that the mouse model is not fully representative of the human state, we do further research by collecting human samples.

### Supplementary Information

Below is the link to the electronic supplementary material.

Supplementary file1 (DOCX 890 KB)

## Abbreviations

CE: Collision energy
CS: Chondroitin sulfate
DMs: Differential metabolites
ECM: Extracellular matrix
GAGs: Glycosaminoglycans
HA: Hyaluronic acid
HS: Heparan sulfate
HRMS: High-resolution mass spectrometry
IDA: Information-dependent acquisition
ISVF: Ion spray voltage floating
KEGG: Kyoto encyclopedia of genes and genomes
MODS: Multi-organ dysfunction syndrome
PCA: Principal component analysis
QC: Quality control
SDC-1: Syndecan-1
