# Supplementary material for: Knockdown of SDC-1 Gene Alleviates the Metabolic Pathway for the Development of MODS
Source: Mol Biotechnol. 2023 Jul 29;66(8):1961–9. doi: 10.1007/s12033-023-00809-9 (PMC11281952; doi:10.1007/s12033-023-00809-9)
Supplement: Supplementary file 1 — Supplementary file1 (DOCX 890 KB) [file 12033_2023_809_MOESM1_ESM.docx]

**
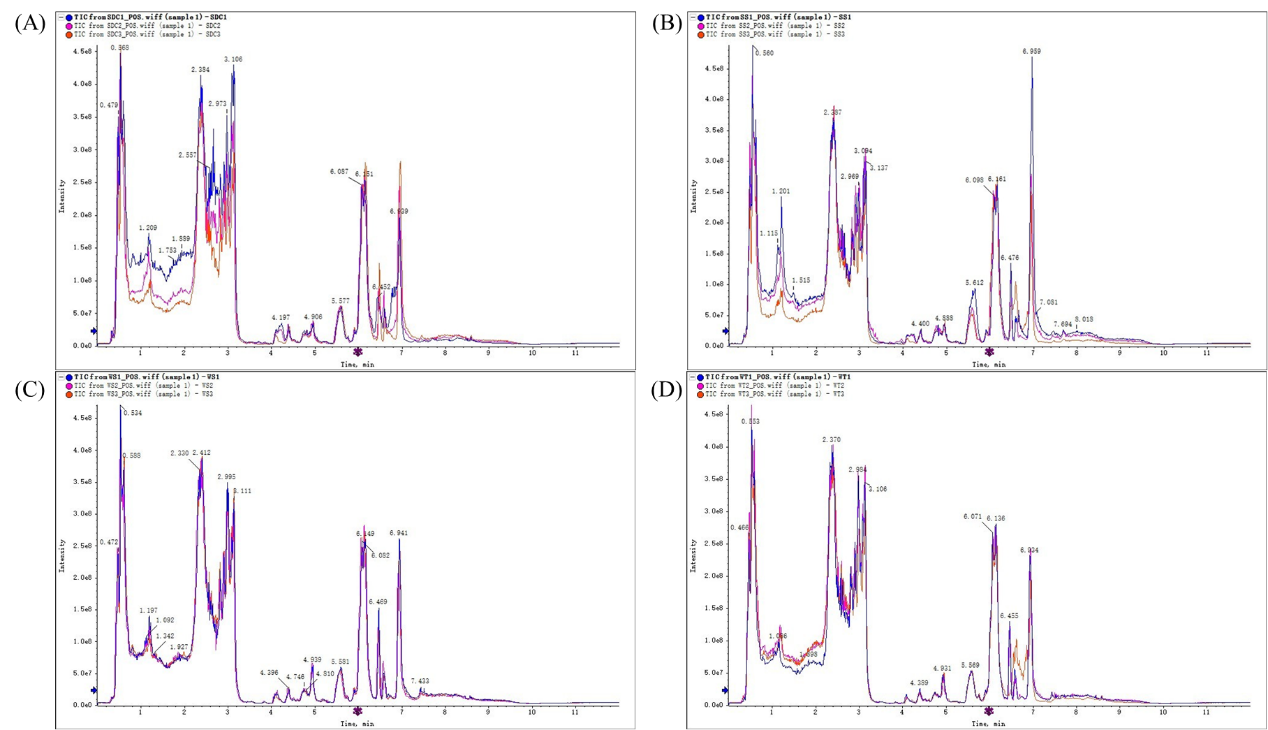
**


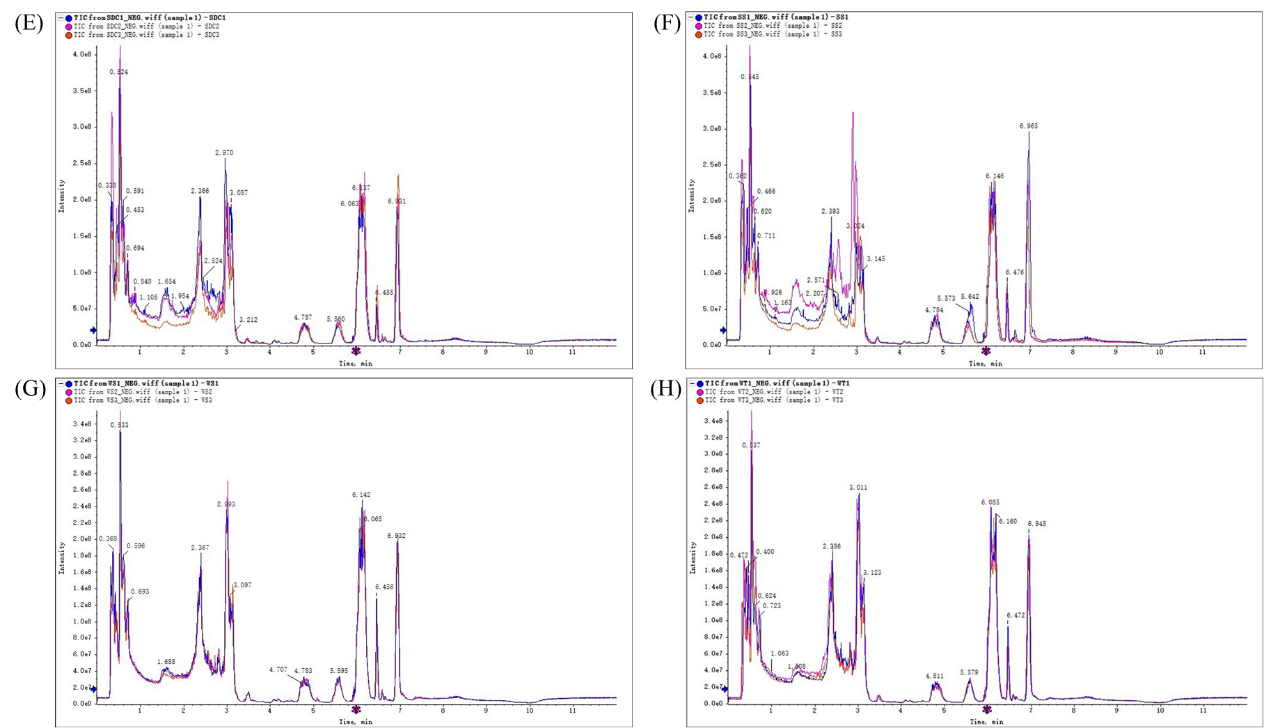


**Figure S1**. Representative total ion chromatograms (TICs) of UPLC-TOF-MS overlaid in negative and positive modes from serum samples. Serum QC samples under positive mode for (A), (B), (C) and (D); Serum QC samples under positive mode for (E), (F), (G) and (H). The different colors represent the chromatographic information of each QC sample.
